# Local Reports of Epidemic and Endemic Diseases

**Published:** 1855

**Authors:** 


### LOCAL REPORTS OF EPIDEMIC AND ENDEMIC DISEASES,

*During the Months of Dec.* 1854, *and Jan. and Feb.* 1855.

The subjoined report is compiled from data furnished specially for this journal by medical men in twelve towns of England and Wales, viz. :—

| Place. | County. | Lat. | Long. | Observer. |
|---|---|---|---|---|
| Hastings . . | Sussex . . | 50.51 N. | 0.34 E. | W. A. Greenhill, M.D. |
| Bridgewater . | Somerset . . | 51. 8 N. | 3. 0 E. | Alfred Haviland, Esq. |
| Canterbury . | Kent . . . | 51·17 N. | 1· 4 W. | G. Rigden, Esq. |
| Putney . . | Surrey . . | 51·28 N. | 0·13 W. | R. H. Whiteman, Esq. |
| Wanstead . . | Essex . . | 51·32 N. | 0· 2 W. | Fred. Collins, M.D. |
| Swansea . . | Glamorganshire . | 51·38 N. | 3·50 W. | W. H. Michael, Esq. |
| Saffron Walden . | Essex . . | 52· 3 N. | 0·12 E. | T. Spurgin, Esq. |
| „ „ | „ . . | „ | „ | H. Stear, Esq. |
| Bedford . . | Bedfordshire . | 52. 8 N. | 0.10.51 W. | T. H. Barker, M.D. |
| Thetford . . | Norfolk . . | 52.26 N. | 0.45 W. | H. W. Bailey, Esq. |
| Nottingham . | Nottinghamshire | 52.30 N. | 1.10 W. | T. Robertson, M.D. |
| Hawarden . | Flintshire . . | 53.11 N. | 3. 2 W. | T. Moffat, M.D. |
| Gainsborough . | Lincolnshire . | 53.23 N. | 0.47 W. | D. Mackinder, M.D. |

Each observer is provided with a printed tabular form, arranged in such a way that weekly records may be kept of the occurrence of each of the following diseases, viz. :—1. Scarlet Fever; 2. Measles; 3. Small-pox; 4. Hooping-cough; 5. Croup; 6. Catarrh; 7. Influenza; 8. Erysipelas; 9. Cholera; 10. Ague; 11. Remittent Fever; 12. Diarrhœa; 13. Dysentery; 14. Typhus; 15. Puerperal Fever; 16. Carbuncle.

### QUARTERLY STATEMENT—No. I.

| Disease. | Where Observed. | At what Periods. |
|---|---|---|
| 1. Scarlatina . , | Hastings . . | Every week. |
| „ | Canterbury . . | Weeks ending from Dec. 27 to Feb. 7 |
| „ | Wanstead . . | Week ending Feb. 28. |
| „ | Swansea . . | From Dec. 27 to Feb. 28. |
| „ | Saffron Walden . | Weeks ending Jan. 17, 24 and 31, and Feb. 7, 14, and 21 |
| „ | Nottingham . . | Weeks ending Jan. 3, 10, 17, and 31, and Feb. 14. |
| „ | Gainsborough . | During whole of January. |
| 2. Measles . . | Bridgewater . . | Weeks ending Feb. 14 and 21. |
| „ | Putney . . | Weeks ending Feb. 14, 21, and 28. |
| „ | Swansea . . | During whole of February. |

| Disease. | Where Observed. | At what Periods. |
|---|---|---|
| 2. Measles . . | Nottingham . . | Weeks ending Feb. 7 and 14. |
| " | Gainsborough . | End of December and begin. of Jan. |
| 3. Small-pox . . | Hastings . . | Week ending Feb. 21. |
| " | Putney . . | Week ending Jan. 31. |
| " | Saffron Walden . | Weeks from Jan. 24 to Feb. 28. |
| " | Bedford . . | Weeks ending Jan. 24 and Feb. 28. |
| " | Nottingham . . | Week ending Feb. 7. |
| 4. Hooping-cough . | Bridgewater . . | During whole of Jan. and Feb. |
| " | Swansea . . | Week ending Feb. 14. |
| " | Saffron Walden . | Week ending Jan. 24. |
| " | Thetford . . | Week ending Jan. 10 |
| " | Nottingham . . | Weeks end. Jan. 10, 17 & 31, & Feb. 28. |
| 5. Croup . . | Hastings . . | Weeks ending Jan. 3, 10, and 24. |
| " | Putney . . | Week ending Dec. 27. |
| " | Wanstead . . | Week ending Jan. 10. |
| " | Swansea . . | Week ending Feb. 14. |
| " | Saffron Walden . | Weeks ending Dec. 27 and Jan. 24. |
| " | Bedford . . | Weeks ending Dec. 27 & Jan. 10 & 17. |
| " | Nottingham . . | Weeks ending Jan. 10 and 17, and whole of February. |
| " | Hawarden . . | Week ending Jan. 10. |
| " | Gainsborough . | Week ending Jan. 24. |
| 6. Catarrh . . | Bridgewater . . | Weekly from Dec. 27. |
| " | Putney . . | Weeks ending from Dec. 27 to Feb. 7. |
| " | Wanstead . . | Weekly from Dec. 27. |
| " | Swansea . . | From Jan. 17 to end of February. |
| " | Saffron Walden . | From Jan. 10 to end of February. |
| " | Bedford . . | During whole of January. |
| " | Thetford . . | Weeks ending from Jan. 24 to Feb. 28. |
| " | Nottingham . . | Whole of January and February. |
| " | Hawarden . . | Weeks ending Dec. 20 and Jan. 10. |
| " | Gainsborough . | Weekly from Dec. 27. |
| 7. Influenza . . | Hastings . . | Weeks ending Jan. 10 & 24, Feb. 7 & 21. |
| " | Bridgewater . . | Whole of January and February. |
| " | Canterbury . . | Whole of January and February. |
| " | Putney . . | Weeks ending Feb. 14, 21, and 28. |
| " | Wanstead . . | December, January, and February. |
| " | Swansea . . | Weeks ending from Jan. 17 to Feb. 28. |
| " | Saffron Walden . | Weeks ending from Jan. 24 to Feb. 28. |
| " | Bedford . . | Weeks ending from Dec. 27 to Feb. 28. |
| " | Thetford . . | Weeks ending from Jan. 17 to Feb. 28. |
| " | Nottingham . . | Whole of January and February. |
| " | Hawarden . . | Weeks ending from Jan. 10 to Feb. 14. |
| " | Gainsborough . | During whole of February. |
| 8. Erysipelas . . | Canterbury . . | Weeks ending Dec. 27, Jan. 17, Feb. 14 |
| " | Putney . . | Weeks ending Jan. 3, Feb. 14 and 28. |
| " | Wanstead . . | Weeks ending Jan. 10 & 17 & Feb. 7. |
| " | Swansea . . | Weeks ending Feb. 21 and 28. |
| " | Saffron Walden . | Weeks ending Jan. 13 and 20. |
| " | Bedford . . | Weeks ending Jan. 3 & 17, Feb. 7 & 14. |

| Disease. | Where Observed. | At what Periods. |
|---|---|---|
| 8. Erysipelas . | Thetford | Week ending Feb. 28. |
| ” | Nottingham | Whole of January and February. |
| ” | Hawarden | Weeks ending Dec. 6 and 13. |
| ” | Gainsborough | Weeks ending Feb. 21 and 28. |
| 9. Cholera | No cases recorded. | |
| 10. Ague | Bridgewater | Week ending Feb. 7. |
| ” | Wanstead | Week ending Jan. 31. |
| ” | Bedford | Weeks ending Jan. 10, 24 & 31, Feb. 14. |
| ” | Thetford | Week ending Feb. 28. |
| 11. Remittent Fever | Swansea | Weeks ending Feb. 14, 21 and 28. |
| ” | Saffron Walden | Weeks ending Feb. 21 and 28. |
| ” | Thetford | Weeks ending Feb. 7 and 28. |
| ” | Nottingham | Weeks ending from Jan. 10 to Feb. 14. |
| ” | Hawarden | Week ending Jan. 24. |
| 12. Diarrhœa . | Bridgewater | Week ending Jan. 24. |
| ” | Wanstead | Weeks ending Dec. 27, Jan. 30, and Feb. 7. |
| ” | Saffron Walden | Weeks ending Dec. 27, Jan. 3, 17 and 31, and Feb. 28. |
| ” | Bedford | Weeks ending Dec. 27, and from Jan. 20 to Feb. 28. |
| ” | Thetford | From Feb. 7 to end of month. |
| ” | Nottingham | During nearly whole of Jan. & Feb. |
| ” | Hawarden | During whole of period. |
| ” | Gainsborough | During Dec. (?), Jan., and Feb. |
| 13. Dysentery . | Wanstead | Week ending Jan. 17. |
| ” | Bedford | Week ending Feb. 14. |
| ” | Nottingham | Weeks ending Jan. 10, 17 and 31, to Feb. 21. |
| ” | Gainsborough | Weeks ending Feb. 14, 21 and 28. |
| 14. Typhus | Hastings | Week ending Dec. 13. |
| ” | Bridgewater | Week ending Feb. 14. |
| ” | Canterbury | Weeks ending from Dec. 27 to Feb. 28. |
| ” | Wanstead | Weeks ending Jan. 31 and Feb. 7. |
| ” | Bedford | Week ending Jan. 10. |
| ” | Thetford | Weeks ending from Jan. 11 to Feb. 28. |
| ” | Nottingham | Weeks ending Jan. 10 & 31 and Feb. 7. |
| ” | Gainsborough | Throughout period. |
| 15. Puerperal Fever. | Swansea | Weeks ending Jan. 31 and Feb. 6. |
| ” | Saffron Walden | Week ending Jan. 31. |
| ” | Nottingham | Week ending Feb. 14. |
| 16. Carbuncle . | Hastings | Weeks ending Jan. 17 and Feb. 14. |
| ” | Bridgewater | Weeks ending Feb. 21 and 28. |
| ” | Canterbury | Weeks ending from Dec. 27 to Feb. 21. |
| ” | Putney | Week ending Jan. 10. |
| ” | Wanstead | Week ending Jan. 10. |
| ” | Thetford | Week ending Jan. 17. |
| ” | Nottingham | Nearly every week in Jan. and Feb. |
| ” | Gainsborough | Weeks from Dec. 27 to Jan. 31. |

### ADDITIONAL OBSERVATIONS.

*Hastings.*—Dr. Greenhill writes, "The only epidemic disease that prevailed in this borough during the three winter months (December, January, and February), was scarlatina, which was of a malignant type, and was not confined to the worst localities. Fifteen cases ended fatally out of a number roughly estimated at more than 200. Scarlatina has not been epidemic in this borough to the same extent since the year 1840."

*Bridgewater.*—Mr. Haviland says, "The influenza that has prevailed has been characterised by the severity of the neuralgic pains which accompanied it. These were especially felt in the lumbar region on the right side of the spine; they were increased by the warmth of the bed, and relieved by aconite and colchicum.

"Jan. 23.—There was no ozone. Diarrhœa occurred among the poor labourers; influenza among men and cattle; jaundice among the latter. Wind N.E. Thermometer varying at 10 A.M. from 28° to 36°. Snow.

"— 30.—No ozone. Wind generally N.E. Neuralgic affections prevalent. Thermometer at 10 A.M. from 32° to 37°.

"Feb. 6.—Snow. Wind E. and S.E. Thermometer at 10 A.M. from 27° to 38°. Amount of ozone for the week, 2.

"— 15.—The river Parrett frozen over for three miles. The amount of ozone present has been in the direct rate of the force of the wind."

*Wanstead.*—Dr. Collins mentions a case of sudden death on January 26th.

*Canterbury.*—Mr. Rigden writes, that scarlet fever has been prevalent in this city during several months. Small-pox prevailed during January and February in the military barracks, but did not reach the city until March.

*Putney.*—Mr. Whiteman states, that influenza and measles had prevailed extensively, but no case under his care had been fatal.

*Swansea.*—Mr. Michael states that the two cases of puerperal fever occurred in females pregnant for the first time, who had suffered great mental anxiety. They were attended by the same nurse; both died. Epidemics, generally of a mild character, were prevalent. The scarlatina was of the simple form; when severe, accompanied by low fever, and in many cases, from exposure to cold, followed by anasarca—and in one case by endocarditis (inflammation of the lining membrane of the heart). The trees were very much injured by the severe winds and intense cold of February.

*Saffron Walden.*—Mr. Spurgin writes: "The cases of small-pox (2) in January, occurred at Inkleton in Cambridgeshire, but were not under my care, and being in an isolated house, did not spread. Cases at Walden, February 10th, were also not under my care, and being concealed by the person, who is said to have brought it from London, it spread to other persons near it, most of whom had been vaccinated. Case under February 28th, was, and is still, under my care; had been vaccinated, but exhibited too large a scar; it has, however, been modified and evidently controlled by vaccination. Scarlet fever was brought from London, but did not extend beyond the family. Catarrh and influenza have been very prevalent. Only one case of puerperal fever has occurred in my practice, which has recovered. Laryngitis has been very prevalent among horses and cattle during the last quarter, and has been very fatal where it has occurred. Vegetation is only just emerging from its dormant state, the earliest spring flowers having made their appearance."

Mr. Stear says, that the cases of scarlet fever occurred principally at Elmdon,* where it had been present since last August. Many of the cases were followed by dropsy; not one fatal case occurred. Catarrh was very prevalent during the better half of January, and the beginning of February.

*Bedford.*—Dr. Barker writes: "Cases of vertigo, parotitis, and bronchitis have been frequent during the quarter. Cases of the following diseases have also occurred, viz.:—pneumonia, chicken-pox, facial neuralgia, lumbago, hæmatemesis, and colic, both with and without vomiting. No case of carbuncle has come under my observation, but cases of whitlow have been frequent."

*Thetford.*—Mr. Bailey says: "The diseases of this quarter have been numerous. The weather during the preceding Autumn was mild, the average temperature being 43°, and the diseases during that period were not of an epidemial origin. The sudden change which took place in temperature from the 10th of January, and the severity of the season, continuing until the middle of March, have occasioned more sickness than we have experienced for some years. Influenza and catarrhal affections were extremely prevalent. Scarcely a family escaped influenza, and with the aged it has been fatal. Many cases of catarrhal affections occurring in families of indigent circumstances, and from the want of common necessaries of life, together with neglect of cleanliness, have assumed a typhoid character, when two or three have occurred in one family; and during the quarter cases occurred every week, thus becoming epidemic. These diseases evidently arise from atmospheric influence, which also produces pneumonia, pleuritis, and bronchitis, especially the latter in children. Five cases of acute rheumatism came under medical treatment during the quarter, and two fatal cases of phthisis pulmonum. I have generally observed that the two extremes of heat and cold produce the same disease. Diarrhœa has been usually prevalent, and might be considered epidemic, in one case almost amounting to cholera, the collapse or prostration being very severe. One case of scarlatina simplex occurred in a young gentleman, who contracted it at school, and was sent home. The severity of the weather, and its continuance to the end of February, the thermometer averaging not more than 33°, may have occasioned the general diseases above recorded. Fevers of a low type have been epidemic,

---

* A village five miles from Walden.

not immediately arising from contagion, as only one of a large family has been attacked under similar positions, while in some families two or three have been affected. Under such circumstances it becomes a debateable point whether the contagious state is specific or only contingent. I am certainly of an opinion that the latter is the most probable. During nearly forty years observations I have attended many cases of one in a large family, poor and indigent, and away from neighbours, and where no other case has occurred, although no precautions were taken at the time.

"The following are the total of Cases from the Registrar's Book :—

| *January.* | | *February.* | |
|---|---|---|---|
| Nephritis | 1 | Typhus | 2 |
| Typhus | 2 | Consumption | 1 |
| Consumption | 3 | Influenza | 3 |
| Bronchitis | 1 | Bronchitis | 1 |
| Broncho-pneumonia | 1 | Peritonitis | 1 |
| Ossification of valves | 1 | Worm fever | 1 |
| Pervious foramen ovale | 1 | Diabetes | 1 |
| Influenza | 2 | Pneumonia | 1 |
| | | Diseased liver | 1 |
| | | Debility | 1 |
| | 12 | | 13 |

Total . . . . 25

"Cases of broncho-pneumonia occurred on the weeks ending January 21st, February 4th, 11th, and 25th, and March 4th and 11th. Cases of bronchitis, weeks ending, January 14th, February 4th, 11th, and 24th, March 4th, 11th, and 18th. Of phthisis, January 14th, 21st, and 28th, February 18th, and March 18th. Of diabetes, January 14th, and February 18th. Of nephrites, January 14th, and February 24th. Of pleuritis, January 24th, February 11th and 18th, and March 11th. Of rheumatitis, January 14th, February 11th and 18th, and March 4th and 18th."

*Nottingham.*—Dr. Robertson writes, "The past quarter is most remarkable for the severe frost of February. All England was under snow after the gale and snow-storm of February 8th. The great snow-storm of January 31st, which occurred in Sussex and Middlesex was scarcely felt here. The river Trent was frozen over nearly a month : generally the ice attained a thickness in still water of seven inches. According to the observations of Mr. Lowe of Highfield House Observatory, the effects of the great cold of 1855 have not been so much felt as that of 1854 ; for although the temperature was nearly as cold as on the 3rd of January, 1854, and although in 1854 the great cold only lasted two or three days, whilst in 1855 it continued for six weeks, still the injury to plants has been, comparatively speaking, slight. The quarter was altogether below the mean of the last forty-two years, except in the beginning of January. The young wheat was somewhat cut. The principal diseases of animals have been acute inflammatory sore-throat, congestive inflammation of the lungs, and disordered action of the liver. Mr. Taylor, the veterinary surgeon of this town, informs me that, in some cases, the laryngeal inflammation has been so great as to necessitate the performance of tracheotomy.

"A reference to the table will shew that influenza and erysipelas and catarrh have been the most prevalent diseases in this neighbourhood. The greatest number of cases commencing on any one day, was on January 21st. The deaths reached a maximum point on January 28th. The number of deaths in January was 146, in February 124. Scarlet fever was fatal in three cases, and carbuncle in one. The wind principally north and north-east. The amount of ozone varied much. It was indicated most constantly between January 28th and February 16th."

*Gainsborough.*—Dr. Mackinder writes,—"I have not had a case of scarlet fever in this town, but have heard of a few cases under other practitioners. In a neighbouring badly drained village, it has been epidemic for some time, and several deaths have occurred. I have had but one case of measles, and one of croup; a few cases of dysentery, and many of diarrhœa. Catarrh and influenza has been epidemic, especially in January, when it affected most those above pauperism. The very severe weather cut off many old people, and a few children, with affections of the chest. Pleuropneumonia has carried off many sheep; and a few young beast have died of what is called the 'black leg.'"

---

